# Chickens productivity selection affects immune system genes

**DOI:** 10.18699/VJ20.670

**Published:** 2020-11

**Authors:** А.М. Borodin, Ya.I. Alekseev, K.E. Gerasimov, N.V. Konovalova, E.V. Тerentjeva, D.N. Efimov, Zh.V. Emanuilova, L.I. Tuchemskiy, A.A. Komarov, V.I. Fisinin

**Affiliations:** Breeding and Genetic Center “Smena”, Bereznyaki, Moscow Region, Russia Institute of Medical and Biological Research, Nizhnii Novgorod, Russia; Limited liability company “Syntol”, Moscow, Russia Institute for Analytical Instrumentation of the Russian Academy of Sciences, St. Petersburg, Russia; Limited liability company “Syntol”, Moscow, Russia; Limited liability company “Syntol”, Moscow, Russia; Limited liability company “Syntol”, Moscow, Russia; Breeding and Genetic Center “Smena”, Bereznyaki, Moscow Region, Russia Federal Scientific Center ”All-Russian Research and Technological Poultry Institute” of the Russian Academy of Sciences, Sergiev Posad, Moscow Region, Russia; Breeding and Genetic Center “Smena”, Bereznyaki, Moscow Region, Russia; Breeding and Genetic Center “Smena”, Bereznyaki, Moscow Region, Russia; Breeding and Genetic Center “Smena”, Bereznyaki, Moscow Region, Russia; Federal Scientific Center ”All-Russian Research and Technological Poultry Institute” of the Russian Academy of Sciences, Sergiev Posad, Moscow Region, Russia

**Keywords:** chickens immune system genes, allele fixation, negative selection, гены иммунной системы кур, фиксация аллелей, негативная селекция

## Abstract

The quantitative trait loci associated with the immune properties of chickens are of interest from the point of view of obtaining animals resistant to infectious agents using marker-assisted selection. In the process of selecting markers for genomic selection in broiler-type chickens, a non-standard genotype frequency of the RACK1 gene allele (SNP Gga_rs15788101) in the B5 line of broiler-type chicken cross Smena 8 was identified and it was suggested that this gene was involved in selection. Therefore, it was decided to investigate the available polymorphisms in the three genes responsible for the IgY titer (DMA, RACK1 and CD1B). Molecular typing of single nucleotide polymorphisms of three loci revealed an approach to fixation of the unfavorable allele of the DMA gene (SNP Gga_rs15788237), an approach to fixation of the unfavorable allele of the RACK1 gene and the prevalence of the favorable CD1B gene allele (SNP Gga_rs16057130). Analysis of the haplotypes revealed a strong linkage disequilibrium of these genes. This suggests that these genes experience selection pressure. Analysis of the protein-coding sequences of the CD1B and DMA genes of various breeds of chickens revealed a negative selection of these genes. In order to understand whether the fixation of the studied alleles is the result of artificial selection of the B5 line of the cross Smena 8, an analysis of similar loci in layer chickens Hisex White was carried out. The frequencies of the alleles at the loci of the CD1B gene (Gga_rs16057130) and the RACK1 gene (Gga_rs15788101) in the Hisex White chicken genome differ from the frequencies of the alleles obtained for chickens of the B5 line of the cross Smena 8. It can be assumed that the fixation of the allele in the DMA gene (SNP Gga_rs15723) is associated with artificial or natural selection, consistent in broilers and layers. Changes in the loci Gga_rs16057130 and Gga_rs15788101 in the B5 line of the Smena 8 chickens are most likely associated with artificial selection of broiler productivity traits, which can subsequently lead to fixation of alleles at these loci. Artificial breeding of chickens leads to degradation of the variability of genes encoding elements of the immune system, which can cause a decrease in resistance to various diseases. The study of the negative impact of selection of economic traits on immunity should provide means to mitigate negative consequences and help find ways to obtain disease-resistant animals.

## Abstract (ru)

Локусы количественных признаков, связанные с иммунными свойствами кур, представляют интерес с точки зрения получения устойчивых к инфекционным агентам животных при помощи маркер- опосредованной (геномной) селекции. В процессе подбора маркеров для геномной селекции у кур брой- лерного типа выявлена нестандартная частота аллеля гена RACK1 (SNP Gga_rs15788101) у кур линии Б5 мясного кросса селекции СГЦ «Смена» и возникло предположение о том, что этот ген вовлечен в селекцию. Поэтому было решено исследовать доступные полиморфизмы в трех генах, ответственных за титр IgY (DMA, RACK1 и CD1B). Молекулярное типирование однонуклеотидных полиморфизмов трех локусов показало при- ближение к фиксации неблагоприятных аллелей генов DMA (SNP Gga_rs15788237) и RACK1 и преобладание благоприятного аллеля гена CD1B (SNP Gga_rs16057130). При анализе гаплотипов выявлено сильное не- равновесное сцепление этих генов. Это свидетельствует о том, что данные гены испытывают селекционное давление. Исследование белок-кодирующих последовательностей генов CD1B и DMA различных пород кур продемонстрировало негативную селекцию этих генов. Для того чтобы понять, является ли фиксация изученных аллелей результатом направленной селекции кур линии Б5 мясного кросса СГЦ «Смена», проведен анализ аналогичных локусов у кур яичной селекции «Хайсекс белый». Частоты аллелей в локусах гена CD1B (Gga_rs16057130) и гена RACK1 (Gga_rs15788101) в геноме кур «Хайсекс белый» отличаются от частот аллелей, полученных для кур линии Б5 мясного кросса селекции СГЦ «Смена». Можно предположить, что фиксация аллеля в гене DMA (SNP Gga_rs15788237) связана с искусственным или естественным отбором, единым для кур мясной и яичной селекции. Изменения в локусах Gga_rs16057130 и Gga_rs15788101 у кур линии Б5 мяс- ного кросса селекции СГЦ «Смена», скорее всего, связаны с искусственной селекцией признаков продуктив- ности бройлеров, которая в дальнейшем может привести к фиксации аллелей в этих локусах. Искусственная селекция кур ведет к деградации вариабельности генов, кодирующих элементы иммунной системы и, как следствие, уменьшению резистентности к различным заболеваниям. Изучение негативного влияния селек- ции хозяйственных признаков на иммунитет должно способствовать снижению отрицательных последствий и поиску способов получения резистентных к заболеваниям животных.

## Introduction

Selecting production traits in the broiler chicken has a negative effect on the breed’s resistance to infectious diseases (Zekarias et al., 2002) and harms their immune competence. On the other hand, resistant chickens are poor producers, e.g. high leucosis resistance in layers reduces egg yield. The only exclusion to this rule has been known so far is resistance to Marek’s disease that stimulates egg laying (Zekarias et al., 2002). One of the studies has demonstrated that broilers produce a short-term humoral response, while layers – a long-term humoral and strong cell-mediated response (Koenen et al., 2002). Another study claims genetic selection to improve the broiler’s growing trait reduces the humoral and increases the cell-mediated and inflammatory responses (Cheema et al., 2003). There are studies proving intensive selection has no negative effect on poultry immune competence (Emam et al., 2014). At the same time, there is a theory saying the resources necessary for physiologically normal immunity are taken to provide the productivity trait in chicken (Zekarias et al., 2002).

After it was found out that antibody titers are genetically inherited, the certain genes affecting this process were detected (Yonash et al., 2001; Kaiser et al., 2002). The quantitative trait loci associated with the immune properties in Gallus gallus belong to different chromosomes (Slawinska, Siwek, 2013; Zhang et al., 2015). A genome-wide search for associations allowed one to detect in chromosomes 1, 3, 5, 12 and 16 nine single-nucleotide polymorphisms (SNPs) in the loci associated with total immunoglobulin Y (IgY) concentration in sera. The most significant five of them are located within a narrow region covering 0.26 Mb of chromosome 16 in the MHC-B locus that determines resistance to viral, bacterial and parasitic infections in chickens. Locus’s variability determines a breed’s resistance to different pathogens (Iglesias et al., 2019). Keeping in mind that the number of haplotypes in the locus is almost one order lower in broilers than in their wild ancestors, it partially may explain the difference in resistance (Nguyen-Phuc et al., 2016). The genes of this region may play a crucial role in immune response modulation (Zhang et al., 2015), so chickens produce IgY to provide their offsprings with an effective humoral response to the most wide-spread pathogens before their own immune system matures (Dias da Silva, Tambourgi, 2010). While choosing genomic selection markers in the parental lines of the B5 line / Smena 8 cross broilers, a nonstandard allele genotype of the RACK1 gene was identified and a suggestion was made that it might be involved in the selection process. For that reason, a decision was made to study the polymorphisms of all the three genes responsible for the IgY titer.

## Materials and methods

To isolate the DNA, feather samples from the 100 broilers belonging to the 79th generation of the B5 line / Smena 8 cross (Cornish breed) bred at Breeding and Genetic Center “Smena” were collected. To isolate the DNA of layer chickens, the feather samples of 48 the Hisex White layer chickens bred at the Zagorskoe Experimental Farm of Federal Scientific Center ”All-Russian Research and Technological Poultry Institute” of the Russian Academy of Sciences were used. The DNA was isolated from the animals’ quill of 0.3–0.5 cm in length as it is required by the investigation protocol for the M-sorb kit (No. HG-501, Syntol LLC, Russia). The PCR required 1.5 μl of isolated DNA and was performed in real time using an АNК-М device (Institute for Analytical Instrumentation of the Russian Academy of Sciences, Russia). The SNPs were typed in two different ways: with the primers containing a modified LNA nucleoside on their 3′ end (Latorra et al., 2003); and introducing two different LNA nucleosides into the 5′ end of a probe in a position compliment to an SNP being studied. While the probe/target mismatch destabilized the interaction, the proper positioning facilitated it. This was due to the presence of LNA modification that significantly changed the thermodynamic characteristics of the samples (You et al., 2006). Using two different channels to detect a fluorescent signal enabled us to detect an SNP in a single test tube, increasing the assay’s effectiveness and simplifying data interpretation. The primer/probe sequences for SNP detection can be seen in Table 1.

**Table 1.**
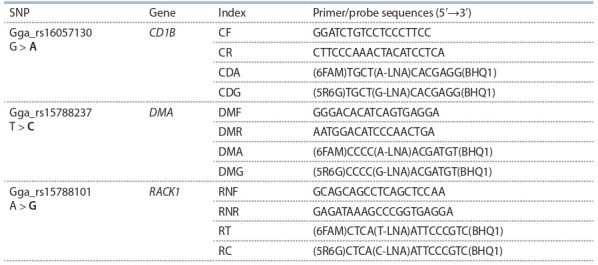
Studied SNPs and their positions in chromosome 16, genes responsible for IgY titers, probes and primers used for the analysis Note. Here and also in Table 2, 4 the nucleolytic sequence enhancing the IgY titer is marked in bold.

**Table 2.**
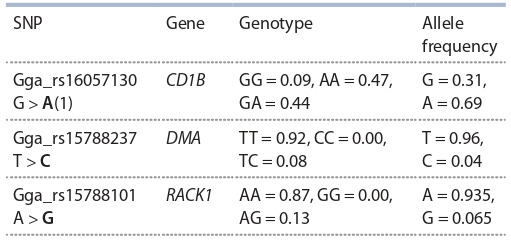
Genotypes and alleles distribution in the B5 line/Smena 8 cross broilers (n = 100)

Such dyes as 6-carboxyfluorescein (6FAM) and 5-carboxyrhodamine 6G (5R6G) were used as fluorescent markers, and the BHQ1 dye – as a quencher. The accumulated data were put through the Ensamble genome browser at https://www.ensembl.org/index.html (Zerbino et al., 2018). The linkage disequilibrium was analyzed using the CubeX webtool (Gaunt et al., 2007) and the DNASp v.6 software (Rozas et al., 2017). The positive and negative selection was analyzed in the HyPhy software (Kosakovsky Pond, Frost, 2005) from the Datamonkey web server (http://datamonkey.org) using the following sequences from GenBank: AB268588.1, AY849318.1, NM_001024582.1, AB204802.1, AY375530.1 for the CD1B gene; AB268588.1, FJ770458.1, NM_001099353.2, HM545127.1, AB426148.1 for the DMA gene; and AY393848.1, M24193.1, NM_001004378.2, CR386189.1, AY694127.1 for the RACK1 gene. Protein secondary structure errors due to mutations were analyzed on the Dim-Pred (Disorder inducing mutation prediction) server at http://www.iitm.ac.in/bioinfo/DIM_Pred/ (Anoosha et al., 2015). The protein 3D structures of corresponding genes were modeled on the SWISS-MODEL web server at http://www.expasy.ch/swissmod/SWISS-MODEL.html (Waterhouse et al., 2018).

## Results and discussion

SNP typing of the three loci responsible for enhanced IgY titer in the B5 line / Smena 8 cross broilers was carried out. All the three SNPs were localized within their corresponding genes. Fixation of the allele Gga_rs15788237 determining the lowest IgY titer in the locus was revealed, as well as that of an unfavorable allele Gga_rs15788101 and the predominance of a favorable allele Gga_rs16057130. The results of SNP typing can be seen in Table 2.

**CD1B gene.** The CD1 proteins are a family that is similar to MHC class I glycoproteins that expose alien and native antigens, so they can be recognized by T-cells (Barral, Brenner, 2007). The ratio of synonymous (dS) and nonsynonymous (dN) mutations in the 5′ coding sequences of the CD1B gene in the chickens of different origin demonstrated a negative selection to present in two regions. The analysis performed using the HyPhy software showed the gene has two codon- alteration regions that result in amino acid replacement (Table 3).

**Table 3.**
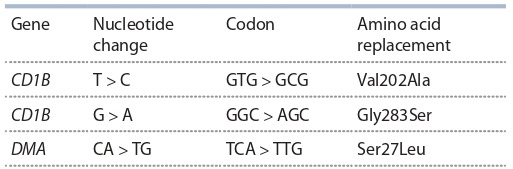
Nucleotide changes in the CD1B and DMA genes, leading to amino acid replacements

However, while replacing nonpolar valine by alanine may not affect the protein’s structure and functions, a replacement of polar serine by nonpolar glycine may significantly alter both the structure and the way the protein interacts with ligands. Analyzing the two mutations on the DIM-Pred server demonstrated their destructive effect on the protein’s secondary structure in which the coding glycine occurred to bind the favorable SNP Gga_rs16057130 allele. 3D modeling of the protein’s structure revealed no visible changes in case of mutual replacements.

**DMA gene.** The DMA gene encodes the alpha-chain of glycoprotein being the receptor to expose alien antigens with specialized T-cells (Chazara et al., 2011). The gene’s dN/dS ratio suggests there is one negative selection site in this gene (see Table 3). The Ser27Leu replacement does not disrupt the protein’s secondary structure but can probably affect the way it interacts with its surrounding and ligands. However, if the site really presents in the DMA gene, a question rises why the unfavorable allele prevails for the IgY titer. A possible explanation can be a balancing selection when a positive selection is substituted by a negative one if the allele frequency becomes high. In this case allele fixation never occurs, so they cannot be regarded either as favorable or unfavorable (Hurst, 2009). In essence, the issue remains open.

**RACK1 gene.** The gene encodes C1, an activated kinase receptor subunit. Analysis of the gene’s dN/dS ratio reveals no signs of the selection determined by the encoding part of the gene, which is hardly a surprise, for this protein is very conservative and 100% match of that in humans. Thus, the hypothetic reason of RACK1 selection remains unidentified and can be predetermined as by the encoding as by the regulation sites in both encoding and non-encoding domains (Chen, Blanchette, 2007; Koonin, Wolf, 2010). Since structural conservation can not be a strong selection factor of its own (Drake et al., 2006; Katzman et al., 2007), several such factors may probably exist.

The allele fixation effect that produces extended runs of homozygosity as a response to environmental and artificial selection factors has recently been found in chickens (Rubin et al., 2010; Fleming et al., 2016). The runs are 3 million base pairs on average and contain a certain number of linked homozygous SNPs (McQuillan et al., 2008; Keller et al., 2011; Hedrick, GarciaDorado, 2016). Finding such runs allows one to detect the genome regions and genes involved in both natural and artificial selections. In tropical climate, adaptation of chickens to traditional poultry production leads to natural selection of birds with favorable genotypes, so the frequency of corresponding alleles increases in the next generations, affecting the homeostasis and immune system genes (Marchesi et al., 2018). For us to understand if the fixation of studied alleles was the result of artificial selection in the B5 line / Smena 8 cross broilers, analysis of similar loci in the Hisex White layer chickens was carried out (Table 4).

**Table 4.**
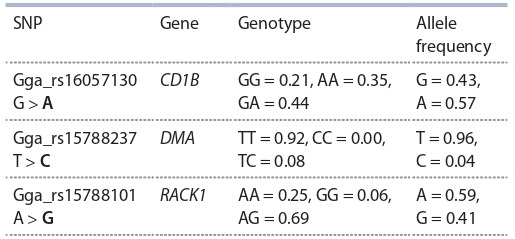
Allele and genotype frequencies distribution in the genome region containing immune system genes in the Hisex White chickens (n = 48)

Similar to the results obtained from the B5 line / Smena 8 chickens, the Hisex White layers had an allele Gga_rs15788237 fixation in the locus. However, the allele frequencies Gga_ rs16057130 and Gga_rs15788101 in the Hisex White genome are different from the frequencies obtained for the B5 / Smena 8 chickens.

Based on the data obtained, an assumption can be made that the allele fixation in DMA gene is related to either artificial or natural selection that is similar for both layer and broiler chickens. The changes in Gga_rs16057130 and Gga_rs15788101 loci observed in the B5 line / Smena 8 cross chickens are most likely related to artificial selection of the productivity traits typical for broilers, which in the future may result in complete allele fixation in these loci. While the fixation, the so-called genetic hitchhiking can take place when an allele changes its frequency not because it is being selected but because it is located next to a gene being selected (Smith, Haigh, 1974; Futuyma, 2013). This phenomenon may involve both favorable and unfavorable alleles of the neighboring gene.

Thus, the presented experiment has confirmed that artificial selection for productivity traits may possibly affect the immune system of chickens, the effect that was first studied by J.J. Li et al. (2017). Typing of the IFIH1 and IFIT5 innate immunity genes has demonstrated an interaction between the production traits and the immune system. It can also be assumed that artificial selection of commercial traits may result in passive selection for immune traits. A study to identify artificial selection regions in chickens (Ma et al., 2018) detected two immunity genes: BCL2L14 (apoptosis mediator) and CDH13 (encoding protein enhancing immune resistivity to Campylobacter jejuni). Resequencing of the cockfighting chicken genome (Guo et al., 2016) detected multiple immunity genes involved in the selection process.

All the authors mentioned above reported about the genes being involved in selection but not directly related to productivity traits. However, they did not mention whether favorable or unfavorable alleles were selected. The fact that unfavorable alleles can be selected as well has long been known since both artificial and natural selection increases the frequency of the rare recessive alleles having a negative effect on viability (Hocking, 2014). This can be exemplified by skeleton and muscle diseases in growing chickens and by multiple ovulations in broilers’ adult parents (Hocking, 2014) meaning the corresponding diseased genes are a part of the parent lines. Other negative manifestations of artificial selections include reduced resistance to infectious disease, pulmonary hypertension and osteoporosis that can be promoted by negative pleiotropic effect or genetic hitchhiking (Elferink et al., 2012). A stark example of such unexpected selection results can be a 22% body mass increase in the chickens bred from crossing the Livorno White lines, which were selected for a long term to produce two-yolk eggs (Abplanalp et al., 1977). All these data prove that selection affects multiple genes. In our study, selection of two unfavorable and one favorable alleles of a single trait was observed, which is important because unfavorable alleles can be beneficial for artificial selection. Forecasting of the unfavourability is based on the variants that greatly affect the phenotype but often turn out to be unstable in nature. However, in artificial conditions, these variants may be quite viable (Hedrick, GarciaDorado, 2016; Bosse et al., 2018).

## Conclusion

Artificial selection in chickens leads to degradation of the variability of the genes encoding the immune system elements that may worsen the birds’ resistance to certain diseases. Studying the negative effect productive trait selection has on the immunity may give us a tool not only to mitigate the effect but also to breed disease-resistant animals.

## Conflict of interest

The authors declare no conflict of interest.

## References

Abplanalp H., Lowry D.C., Van Middelkoop J.H. Selection for increased
incidence of double-yolked egg in white leghorn chickens. Br. Poult.
Sci. 1977;18(5):585-595. DOI 10.1080/00071667708416407.

Anoosha P., Sakthivel R., Gromiha M.M. Prediction of protein disorder
on amino acid substitutions. Anal. Biochem. 2015;491:18-22. DOI
10.1016/j.ab.2015.08.028.

Barral D.C., Brenner M.B. CD1 antigen presentation: how it works.
Nat. Rev. Immunol. 2007;7(12):929-941. DOI 10.1038/nri2191.

Bosse M., Megens H.J., Derks M.F.L., de Cara Á.M.R., Groenen
M.A.M. Deleterious alleles in the context of domestication, inbreeding,
and selection. Evol. Appl. 2018;12(1):6-17. DOI 10.1111/
eva.12691.

Chazara O., Tixier-Boichard M., Morin V., Zoorob R., Bed’hom B. Organisation
and diversity of the class II DM region of the chicken
MHC. Mol. Immunol. 2011;48(9-10):1263-1271. DOI 10.1016/
j.molimm.2011.03.009.

Cheema M.A., Qureshi M.A., Havenstein G.B. A comparison of the
immune response of a 2001 commercial broiler with a 1957 randombred
broiler strain when fed representative 1957 and 2001
broiler diets. Poult. Sci. 2003;82(10):1519-1529. DOI 10.1093/ps/
82.10.1519.

Chen H., Blanchette M. Detecting non-coding selective pressure in
coding regions. BMC Evol. Biol. 2007;7(Suppl 1):S9. DOI 10.1186/
1471-2148-7-S1-S9.

Dias da Silva W., Tambourgi D.V. IgY: a promising antibody for use
in immunodiagnostic and in immunotherapy. Vet. Immunol. Immunophatol.
2010;135(3-4):173-180. DOI 10.1016/j.vetimm.2009.12.
011.

Drake J.A, Bird C., Nemesh J., Thomas D.J., Newton-Cheh C., Reymond
A., Excoffier L., Attar H., Antonarakis S.E., Dermitzakis E.T.,
Hirschhorn J.N. Conserved noncoding sequences are selectively
constrained and not mutation cold spots. Nat. Genet. 2006;38(2):
223-227. DOI 10.1038/ng1710.

Elferink M.G., Megens H.J., Vereijken A., Hu X., Crooijmans R.P.,
Groenen M.A. Signatures of selection in the genomes of commercial
and non-commercial chicken breeds. PLoS One. 2012;7(2):e32720.
DOI 10.1371/journal.pone.0032720.

Emam M., Mehrabani-Yeganeh H., Barjesteh N., Nikbakht G., Thompson-
Crispi K., Charkhkar S., Mallard B. The influence of genetic
background versus commercial breeding programs on chicken immunocompetence.
Poult. Sci. 2014;93(1):77-84. DOI 10.3382/ps.
2013-03475.

Fleming D.S., Koltes J.E., Markey A.D., Schmidt C.J., Ashwell C.M.,
Rothschild M.F., Persia M.E., Reecy J.M., Lamont S.L. Genomic
analysis of Ugandan and Rwandan chicken ecotypes using a 600 K
genotyping array. BMC Genom. 2016;17:407. DOI 10.1186/s12864-
016-2711-5.

Futuyma D.J. Evolution: Third Edition. Sunderland, MA: Sinauer Associates,
Inc., 2013. 656 p.

Gaunt T.R., Rodrigues S., Day I.N. Cubic exact solutions for the estimation
of pairwise haplotype frequencies: implications for linkage
disequilibrium analyses and a web tool ‘CubeX’. BMC Bioinform.
2007;8:428. DOI 10.1186/1471-2105-8-428.

Guo X., Fang Q., Ma C., Zhou B., Wan Y., Jiang R. Whole-genome
resequencing of Xishuangbanna fighting chicken to identify signatures
of selection. Genet. Sel. Evol. 2016;48(1):62. DOI 10.1186/
s12711-016-0239-4.

Hedrick P.W., Garcia-Dorado A. Understanding Inbreeding Depression,
Purging, and Genetic Rescue. Trends Ecol. Evol. 2016;31(12):
940-952. DOI 10.1016/j.tree.2016.09.005.

Hocking P.M. Unexpected consequences of gevnetic selection in broilers
and turkeys: problems and solutions. Br. Poult. Sci. 2014;55(1):1-12.
DOI 10.1080/00071668.2014.877692.

Hurst L.D. Fundamental concepts in genetics: genetics and the understanding
of selection. Nat. Rev. Genet. 2009;10(2):83-93. DOI
10.1038/nrg2506.

Iglesias G.M., Canet Z.E., Cantaro H., Miquel M.C., Melo J.E., Miller
M.M., Berres M.E., Fulton J.E. Mhc-B haplotypes in “Campero-
Inta” chicken synthetic line. Poult. Sci. 2019;98(11):5281-5286.
DOI 10.3382/ps/pez431.

Kaiser M.G., Deeb N., Lamont S.J. Microsatellite markers linked to
Salmonella enterica serovar enteritidis vaccine response in young F1
broiler-cross chicks. Poult. Sci. 2002;81(2):193-201. DOI 10.1093/
ps/81.2.193.

Katzman S., Kern A.D., Bejerano G., Fewell G., Fulton L., Wilson R.K.,
Salama S.R., Haussler D. Human genome ultraconserved elements
are ultraselected. Science. 2007;317(5840):915. DOI 10.1126/
science.1142430.

Keller M.C., Visscher P.M., Goddard M.E. Quantification of inbreeding
due to distant ancestors and its detection using dense single nucleotide
polymorphism data. Genetics. 2011;189(1):237-249. DOI
10.1534/genetics.111.130922.

Koenen M.E., Boonstra-Blom A.G., Jeurissen S.H. Immunological
differences between layer and broiler type chickens. Vet. Immunol.
Immunophatol. 2002;89(1-2):47-56. DOI 10.1016/S0165-2427(02)
00169-1.

Koonin E.V., Wolf Y.I. Constraints and plasticity in genome and molecular-
phenome evolution. Nat. Rev. Genet. 2010;11(7):487-498.
DOI 10.1038/nrg2810.

Kosakovsky Pond S.L., Frost S.D. Not so different after all: a comparison
of methods for detecting amino acid sites under selection.
Mol. Biol. Evol. 2005;22(5):1208-1222. DOI 10.1093/molbev/
msi105.

Latorra D., Campbell K., Wolter A., Hurley J.M. Enhanced allele-specific
PCR discrimination in SNP genotyping using 3′ locked nucleic
acid (LNA) primers. Hum. Mutat. 2003;22(1):79-85. DOI 10.1002/
humu.10228.

Li J.J., Wang Y., Yang C.W., Ran J.S., Jiang X.S., Du H.R., Hu Y.D.,
Liu Y.P. Genotypes of IFIH1 and IFIT5 in seven chicken breeds indicated
artificial selection for commercial traits influenced antiviral
genes. Infect. Genet. Evol. 2017;56:54-61. DOI 10.1016/j.meegid.
2017.10.019.

Ma Y., Gu L., Yang L., Sun C., Xie S., Fang C., Gong Y., Li S. Identifying
artificial selection signals in the chicken genome. PLoS One.
2018;13(4):e0196215. DOI 10.1371/journal.pone.0196215.

Marchesi J.A.P., Buzanskas M.E., Cantão M.E., Ibelli A.M.G., Peixoto
J.O., Joaquim L.B., Moreira G.C.M., Godoy T.F., Sbardella A.P.,
Figueiredo E.A.P., Coutinho L.L., Munari DP., Ledur M.C. Relationship
of runs of homozygosity with adaptive and production traits in a
paternal broiler line. Animal. 2018;12(6):1126-1134. DOI 10.1017/
S1751731117002671.

McQuillan R., Leutenegger A.L., Abdel-Rahman R., Franklin C.S.,
Pericic M., Barac-Lauc L., Smolej-Narancic N., Janicijevic B., Polasek
O., Tenesa A., Macleod A.K., Farrington S.M., Rudan P., Hayward
C., Vitart V., Rudan I., Wild S.H., Dunlop M.G., Wright A.F.,
Campbell H., Wilson J.F. Runs of Homozygosity in European Populations.
Am. J. Hum. Genet. 2008;83(3):359-372. DOI 10.1016/
j.ajhg.2008.08.007.

Nguyen-Phuc H., Fulton J.E., Berres M.E. Genetic variation of major
histocompatibility complex (MHC) in wild Red Junglefowl (Gallus
gallus). Poult. Sci. 2016;95(2):400-411. DOI 10.3382/ps/pev364.

Rozas J., Ferrer-Mata A., Sánchez-DelBarrio J.C., Guirao-Rico S., Librado
P., Ramos-Onsins S.E., Sánchez-Gracia A. DnaSP 6: DNA Sequence
Polymorphism Analysis of Large Data Sets. Mol. Biol. Evol.
2017;34(12):3299-3302. DOI 10.1093/molbev/msx248.

Rubin C.-J., Zody M.C., Eriksson J., Meadows J.R., Sherwood E.,
Webster M.T., Jiang L., Ingman M., Sharpe T., Ka S., Hallböök F.,
Besnier F., Carlborg Ö., Bed’hom B., Tixier-Boichard M., Jensen P.,
Siegel P., Lindblad-Toh K., Andersson L. Whole-genome resequencing
reveals loci under selection during chicken domestication.
Nature.
2010;464(7288):587-591. DOI 10.1038/nature08832.

Slawinska A., Siwek M. Meta - and combined - QTL analysis of different
experiments on immune traits in chickens. J. Appl. Genet. 2013;
54(4):483-7. DOI 10.1007/s13353-013-0177-6.

Smith J.M., Haigh J. The hitch-hiking effect of a favorable gene. Genet.
Res. 1974;23(1):23-35. DOI 10.1017/S0016672308009579.

Waterhouse A., Bertoni M., Bienert S., Studer G., Tauriello G., Gumienny
R., Heer F.T., de Beer T.A.P., Rempfer C., Bordoli L., Lepore R.,
Schwede T. SWISS-MODEL: homology modelling of protein
structures and complexes. Nucleic Acids Res. 2018;46(W1):W296-
W303. DOI 10.1093/nar/gky427.

Yonash N., Cheng H.H., Hillel J., Heller D.E., Cahaner A. DNA microsatellites
linked to quantitative trait loci affecting antibody response
and survival rate in meat-type chickens. Poult. Sci. 2001;80(1):22-
28. DOI 10.1093/ps/80.1.22.

You Y., Moreira B.G., BehLke M.A., Owczarzy R. Design of LNA
probes that improve mismatch discrimination. Nucleic Acids Res.
2006;34(8):e60. DOI 10.1093/nar/gkl175.

Zekarias B., Ter Huurne A.A., Landman W.J., Rebel J.M., Pol J.M.,
Gruys E. Immunological basis of differences in disease resistance
in the chicken. Vet. Res. 2002;33(2):109-125. DOI 10.1051/vetres:
2002001.

Zerbino D.R., Achuthan P., Akanni W., Amode M.R., Barrell D., Bhai J.,
Billis K., Cummins C., Gall A., Girón C.G., Gil L., Gordon L., Haggerty
L., Haskell E., Hourlier T., Izuogu O.G., Janacek S.H., Juettemann
T., To J.K., Laird M.R., Lavidas I., Liu Z., Loveland J.E., Maurel
T., McLaren W., Moore B., Mudge J., Murphy D.N., Newman V.,
Nuhn M., Ogeh D., Ong C.K., Parker A., Patricio M., Riat H.S.,
Schuilenburg H., Sheppard D., Sparrow H., Taylor K., Thormann A.,
Vullo A., Walts B., Zadissa A., Frankish A., Hunt S.E., Kostadima
M., Langridge N., Martin F.J., Muffato M., Perry E., Ruffier M.,
Staines D.M., Trevanion S.J., Aken B.L., Cunningham F., Yates A.,
Flicek P. Ensembl 2018. Nucleic Acids Res. 2018;46(D1):D754-
D761. DOI 10.1093/nar/gkx1098.

Zhang L., Li P., Liu R., Zheng M., Sun Y., Wu D., Hu Y., Wen J., Zhao G.
The identification of loci for immune traits in chickens using a genome-
wide association study. PloS One. 2015;10(3):e0117269. DOI
10.1371/journal.pone.0117269.

